# Hydrothermal Growth of Vertically Aligned ZnO Nanorods Using a Biocomposite Seed Layer of ZnO Nanoparticles

**DOI:** 10.3390/ma6083584

**Published:** 2013-08-19

**Authors:** Zafar Hussain Ibupoto, Kimleang Khun, Martin Eriksson, Mohammad AlSalhi, Muhammad Atif, Anees Ansari, Magnus Willander

**Affiliations:** 1 Physical Electronics and Nanotechnology Division, Department of Science and Technology, Campus Norrköping, Linköping University, SE-60174 Norrköping, Sweden; E-Mails: kimleang.khun@liu.se (K.K.); magnus.willander@liu.se (M.W.); 2 Department of Physics, Chemistry, and Biology (IFM), Linköping University, 58183 Linköping, Sweden; E-Mail: marer@ifm.liu.se; 3 Physics and Astronomy Department, College of Science, King Saud University, 11451 Riyadh, Saudi Arabia; E-Mail: atifhull@gmail.com; 4 Research Chair for Laser Diagnosis of Cancer, King Saud University, 11451 Riyadh, Saudi Arabia; E-Mail: malsalhi@ksu.edu.sa; 5 King Abdullah Institute for Nanotechnology, King Saud University, 11451 Riyadh, Saudi Arabia; E-Mail: aneesaansari@gmail.com

**Keywords:** biocomposite seed layer, ZnO nanorods, hydrothermal growth method, starch, cellulose

## Abstract

Well aligned ZnO nanorods have been prepared by a low temperature aqueous chemical growth method, using a biocomposite seed layer of ZnO nanoparticles prepared in starch and cellulose bio polymers. The effect of different concentrations of biocomposite seed layer on the alignment of ZnO nanorods has been investigated. ZnO nanorods grown on a gold-coated glass substrate have been characterized by X-ray diffraction (XRD) and field emission scanning electron microscopy (FESEM) techniques. These techniques have shown that the ZnO nanorods are well aligned and perpendicular to the substrate, and grown with a high density and uniformity on the substrate. Moreover, ZnO nanorods can be grown with an orientation along the *c*-axis of the substrate and exhibit a wurtzite crystal structure with a dominant (002) peak in an XRD spectrum and possessed a high crystal quality. A photoluminescence (PL) spectroscopy study of the ZnO nanorods has revealed a conventional near band edge ultraviolet emission, along with emission in the visible part of the electromagnetic spectrum due to defect emission. This study provides an alternative method for the fabrication of well aligned ZnO nanorods. This method can be helpful in improving the performance of devices where alignment plays a significant role.

## 1. Introduction

During the last two decades, a great deal of research has been performed on the synthesis of different nanostructures, which helps in the understanding of the mesophysics phenomena of growing nonmaterial. Several nanostructures of different metal oxide semiconductors with potential applications have been explored. Among these, ZnO is considered to be a promising material for the nanoscale based device applications due to its wurtzite crystal structure, wide direct band gap of 3.37 eV and high exciton binding energy of 60 meV. ZnO nanostructures can be used in the development of light emitting diodes (LEDs) [1], piezoelectric transducers [2,3], gas sensors [4,5], dye-sensitized solar cells [6], and in medical applications [7]. As research on the fabrication and applications of ZnO is maturing, the pronounced effect of the morphology of ZnO nanostructures has been debated. It is crucial to have a controlled morphology of the nanostructures for the desired application, due to its backbone role in the performance of the device. Several methods have been reported for the synthesis of various ZnO nanostructures, both with physical and chemical approaches. Yet, more work is required in the synthesis of well align ZnO nanostructures. The oriented ZnO morphology is highly demanded for the construction of devices. Among the diverse morphologies of ZnO, nanorods have gained particular attention in the research community. As a result, several approaches exist for their fabrication. To grow one dimensional ZnO nanostructures, such as nanorods, many expensive growth techniques are available, including pulsed laser deposition (PLD) [8], thermal evaporation [9], vapor transport deposition (VTD) [10], molecular beam epitaxy (MBE) [11], different chemical vapor deposition (CVD) techniques [12,13,14], and magnetron sputtering (MS) [15]. These methods require extremely severe growth conditions, including high temperature and pressure, and dangerous chemicals. Besides these, low cost and simple growth techniques, such as electrodeposition [16] and hydrothermal growth methods [17], can be used for the fabrication of one dimensional ZnO nanostructures. The seed-free growth of highly ordered ZnO nanorods is very difficult on many substrates, because of lattice mismatch. A seed layer is therefore essential in order to achieve well aligned nanorods on the substrate. Recently, several seed coating techniques have been used for the growth of well-ordered ZnO nanorods, such as atomic layer deposition (ALD), pulsed laser deposition (PLD) [18], electron beam evaporation (EBE) [19], the successive ionic layer adsorption and reaction (SILAR) method [20], spray pyrolysis [21], and RF sputtering techniques [22,23]. The growth of ZnO nanorods, homogeneously perpendicular to the substrate, is a challenging task using a simple and low temperature aqueous chemical growth method. Moreover, template assisted methods have also been used for their potential capability in the development of ordered materials in a controlled way [24,25]. The template assisted materials include micelles [26], membranes [27], biopolymers [28,29], as well as animal and plant tissues [30,31].

In this work, freshly prepared ZnO nanoparticles were homogenized with two renowned biopolymers; starch and cellulose. The biopolymer solutions were used as a seed layer for the synthesis of highly ordered ZnO nanorods on the gold coated glass substrates. Both starch and cellulose are among the most abundant naturally occurring biopolymers, and starch has the ability to form a variety of complexes with other molecules [32]. Starch is a polymer formed by repeating units of amylose and amylopectin through 1, 4 glycoside linkages between D-glucose units. Starch is not easily soluble in water at room temperature, but by heating the water, it forms a gelatin liquid. In this gelatin liquid, ZnO nanoparticles provide a large number of nucleation sites for the growth of well aligned ZnO nanorods. Similarly, cellulose is also a member of the starch family, and being hydrophilic in nature, it provides a useful platform for distributing ZnO nanoparticles on the substrate and thereby acting as an efficient seed material together with ZnO nanoparticles for the fabrication of well aligned ZnO nanorods. In addition to this, the effect of different concentrations of ZnO nanoparticles diffused in starch and cellulose biopolymers on the alignment of ZnO nanorods have been investigated.

## 2. Results and Discussion

**Figure 1:**
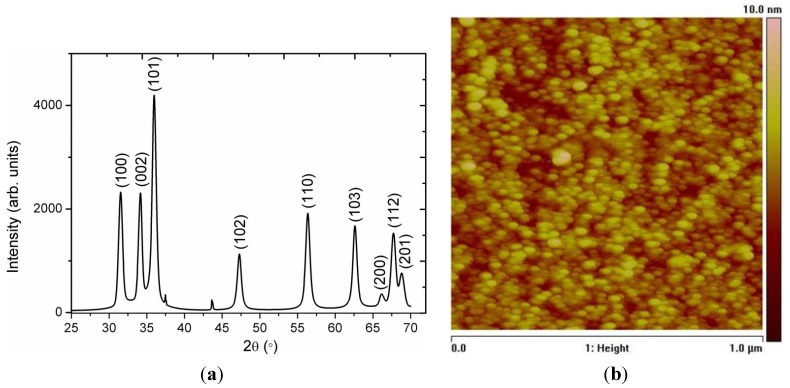
(**a**) The XRD pattern of ZnO nanoparticles; (**b**) The AFM image of the biocomposite seed layer of ZnO nanoparticles.

ZnO nanoparticles were characterized X-ray diffraction (XRD) technique and the diffraction pattern is shown in Figure 1a. The measured diffraction peaks include 100, 002, 101, 102, 110, 103, 200, 112, and 201, which are according to the reported work [33]. The ZnO nanoparticles exhibited the nanocrystalline phase and no other impurity such as Zn(OH)_2_ was observed. Figure 1b shows the AFM image of the biocomposite seed layer of the ZnO nanoparticles and it can be seen that the nanoparticles are uniform, dense and well adhered to the gold-coated glass substrate which behaved better nucleation centers for the growth of well aligned ZnO nanorods.

**Figure 2:**
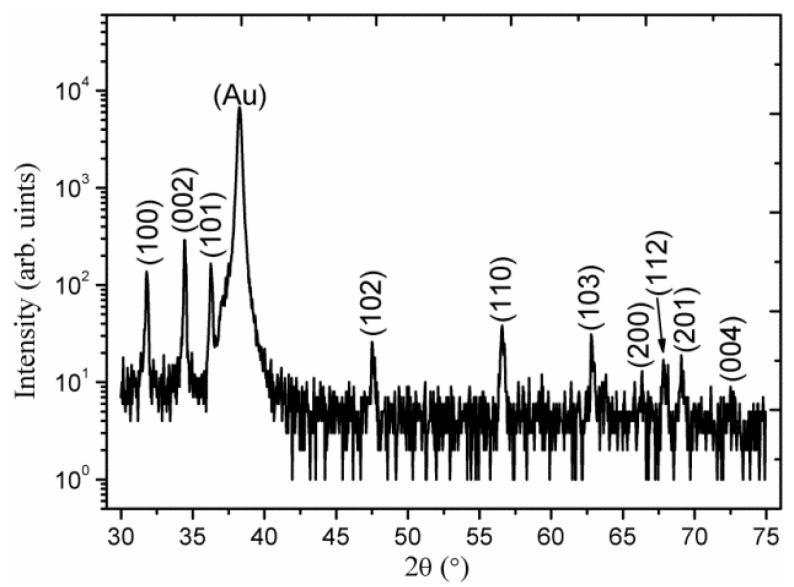
The XRD pattern of ZnO nanorods without seed solution.

**Figure 3:**
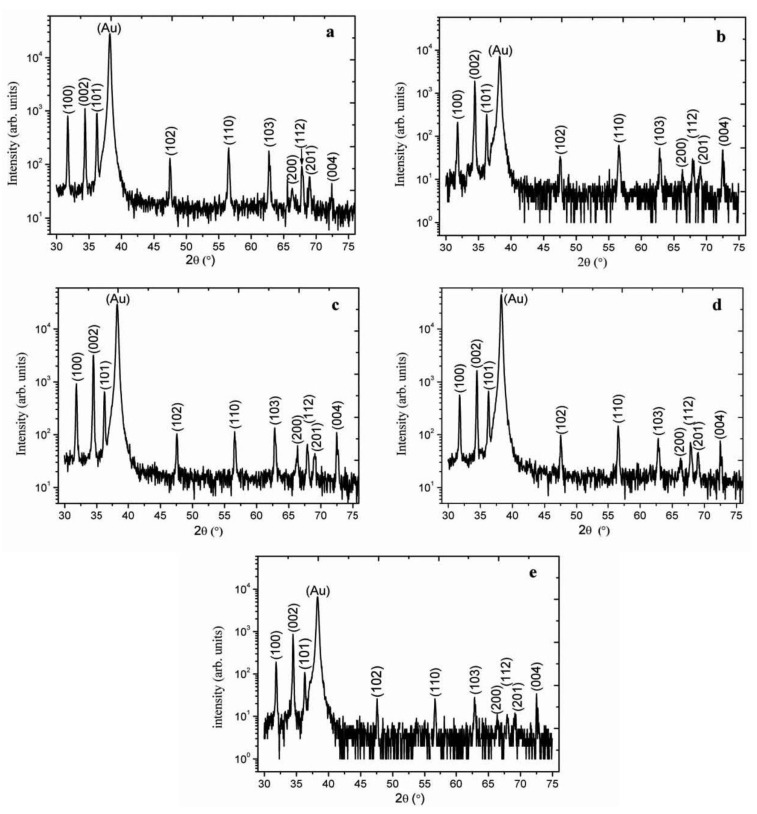
The XRD pattern of ZnO nanorods growth with seed solutions containing 3.5 mg/mL of starch concentration and different concentrations of ZnO nanoparticles: (**a**) 0.5; (**b**) 1.0; (**c**) 1.5; (**d**) 2.0; and (**e**) 2.5 mg/mL.

**Figure 4:**
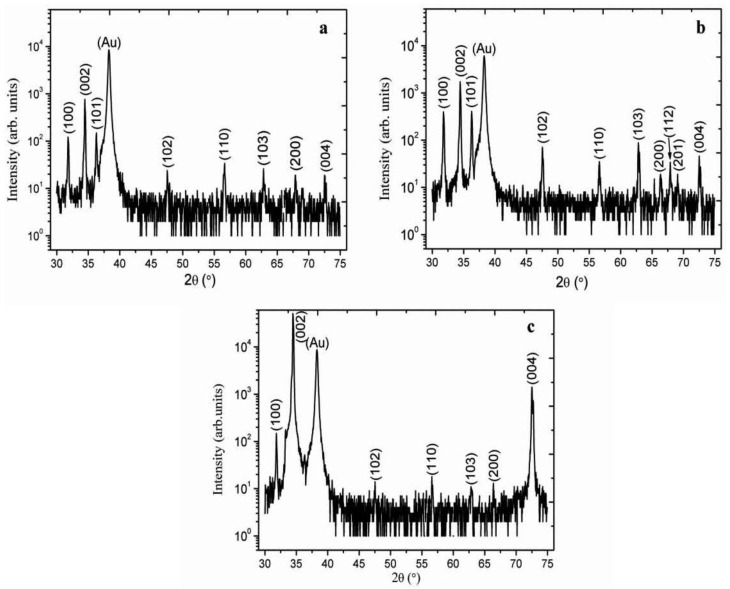
The XRD pattern of ZnO nanorods growth with seed solutions containing 3.5 mg/mL of cellulose concentration with different concentrations of ZnO nanoparticle: (**a**) 1.0; (**b**) 3.5; and (**c**) 7.5 mg/mL.

Figure 2 shows the XRD spectra of as synthesized ZnO nanorods without the seed layer of ZnO nanoparticles and it can be observed that the appeared peaks are less intense especially (002) peak which proved that without seed layer the orientation of nanorods is subject of matter. However, with the seed layer consisting of ZnO nanoparticles in starch biocomposite as shown in Figure 3a–e, the measured diffraction peaks are almost similar, but the orientation is a bit improved. For cellulose biocomposite based seed layer, on gold-coated substrates, the observed 2θ peaks at 31.7°, 34.4°, 36.3°, 47.5°, 56.6°, 62.8°, 67.9°, and 72.5° are shown in Figure 4a–c. All the obtained XRD diffraction patterns are according to JCPDS card number 80-0075. The peaks in the XRD spectrum could be assigned to the (100), (002), (101), (102), (110), (103), (112), and (004) crystal planes of ZnO nanorods with wurtzite crystal structures. The nanorods grown on a starch seed layer, and in particular those grown on a cellulose seed layer, are well-ordered due to very intense peak of (002) crystal plane which shows that the growth pattern is along the *c*-axis direction as shown in Figure 4c.

**Figure 5:**
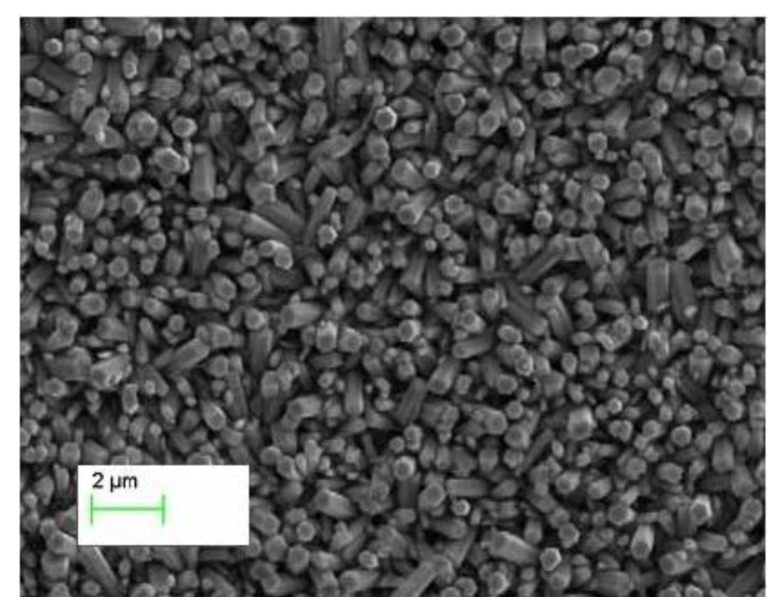
The FESEM image of ZnO nanorods grown with only ZnO nanoparticles seed solution.

**Figure 6:**
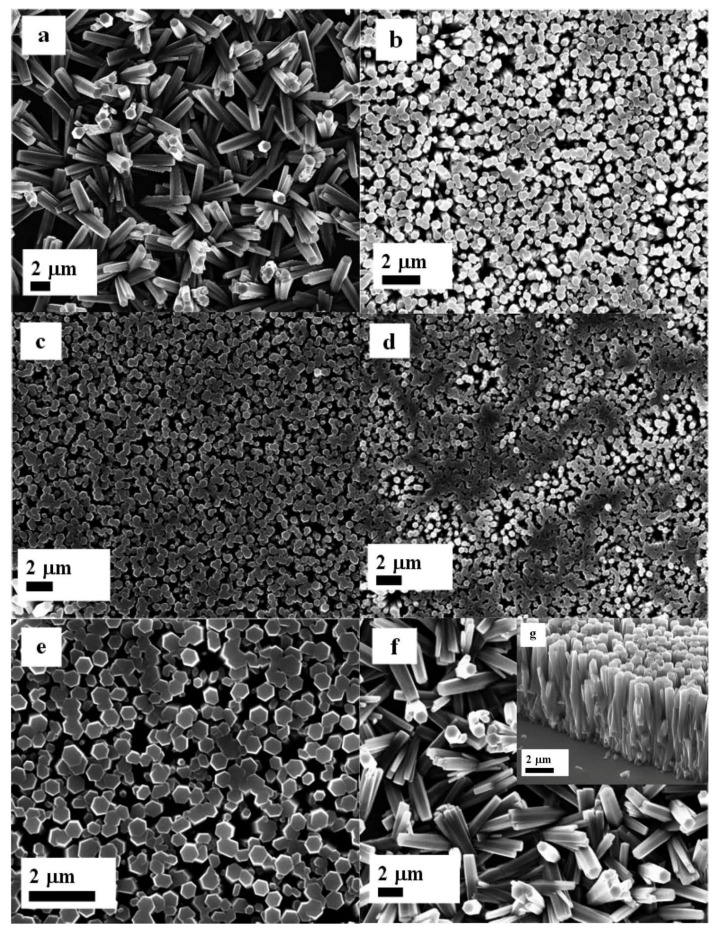
The FESEM images of ZnO nanorods with seed solutions containing 3.5 mg/mL of starch concentration and (**a**) 0; (**b**) 0.5; (**c**) 1.0; (**d**) 1.5; (**e**) 2.0; (**f**) 2.5 mg/mL of ZnO nanoparticles concentration; and (**g**) cross section image of ZnO nanorods growth with f condition.

**Figure 7:**
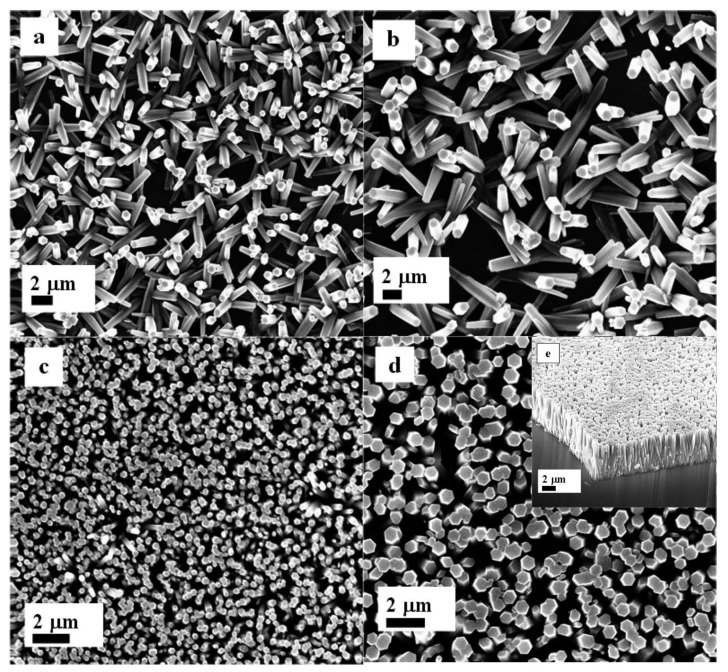
The FESEM images of ZnO nanorods with seed solutions containing 3.5 mg/mL of cellulose concentration with difference amount: (**a**) 0; (**b**) 1.0; (**c**) 3.5; (**d**) 7.5 mg/mL of ZnO nanoparticles concentration; and (**e**) cross section image of ZnO nanorods growth with d condition.

A distinctive FESEM image of ZnO nanorods grown with the seed layer of ZnO nanoparticles (without starch and cellulose) is shown in Figure 5 and it can be seen that to some extent the nanorods are aligned and exhibited uniform diameter which could be due to the improper nucleation on the substrate provided by ZnO nanoparticles and possible the aggregation of nanoparticles. Typical FESEM images of the ZnO nanorods growth on seed layers of ZnO nanoparticles biocomposite with starch and cellulose on the gold-coated glass substrates are shown in Figure 6 and Figure 7 respectively.

Figure 6a–g shows the ZnO nanorods grown with seed layer of ZnO nanoparticles and starch biopolymer, Figure 6a is an image of ZnO nanorods grown in a starch biopolymer without ZnO nanoparticles. It can be seen that the ZnO nanorods are not well ordered and the yield of nanorods on the substrate is very much less. However, with the addition of 0.5 mg/mL of ZnO nanoparticles in the starch solution, the grown nanorods are well aligned along the *c*-axis direction of the substrate as shown in Figure 6b. Further increasing the concentration of ZnO nanoparticles to 1.0, 1.5, and 2.0 mg/mL in the seed solution which resulted well-ordered ZnO nanorods, as shown in Figure 6c–e. This behavior can be explained by the gelatin like property of starch and the uniform distribution of ZnO nanoparticles on the surface of the starch biopolymer, which provides the possible nucleation for the growth of ZnO nanorods. However, by further increasing the concentration of ZnO nanoparticles to 2.5 mg/mL in the starch solution, the nanoparticles might start to aggregate on the surface of the starch biopolymer. The aggregation of ZnO nanoparticles in starch results in a loss of uniformity of the distribution of the ZnO nanorods, as well as a random alignment of the nanorods, as shown in Figure 6f.

**Figure 8:**
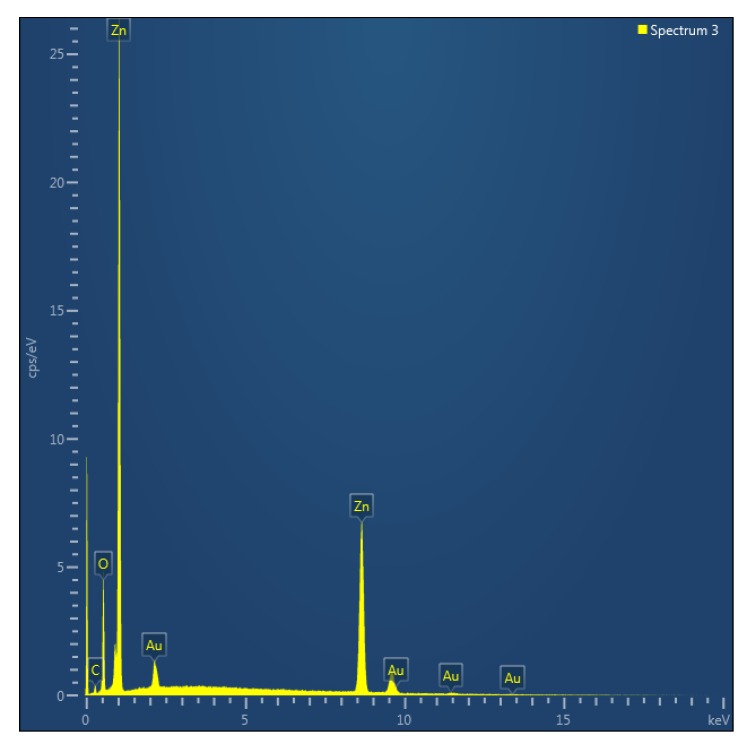
The EDX of ZnO nanorods.

Figure 7a–e shows top view FESEM images of ZnO nanorods fabricated with a cellulose biopolymer seed layer. It can be seen from Figure 7a that omitting the ZnO nanoparticles in the seed layer results in nanorods that grow with larger rod to rod separations and a random alignment. When 1.0 mg/mL of ZnO nanoparticles were introduced in the cellulose solution, the nanorods started to grow in random directions, as shown in Figure 7b. However, for seed layer containing 3.5 and 7.5 mg/mL of ZnO nanoparticles in cellulose solution, the ZnO nanorods are grown highly ordered and dense on the substrate, as shown in Figure 7c,d. Cellulose, being a member of the starch family, shows similar effects on the alignment of the ZnO nanorods as starch does. The FESEM study demonstrates that ZnO nanorods synthesized by a seed layer of ZnO nanoparticles with biopolymers exhibit a hexagonal crystal structure and are oriented along the *c*-axis of the substrate, which is consistent with the XRD results. The diameters of the nanorods, grown by a combination of organic and inorganic materials in the seed layer, are in the range of 100 nm to 200 nm. In other words, the deposition of biocomposite seed layer of ZnO nanoparticles not only assists to control the yield of nanorods, diameter, and their uniform distribution, but also enhances the overall alignment orientation of ZnO nanorods. The EDX technique was used for the study of composition of ZnO nanorods and the obtained results indicate that the prepared sample is only composed of Zn and O atoms as shown in Figure 8.

Photoluminescence (PL) is the efficient tool to study the optical, electronic and structural characteristics of different materials. PL study was carried out for the investigation of defect states and crystal quality of fabricated ZnO nanorods using biocomposite seed layer of ZnO nanoparticles.

**Figure 9:**
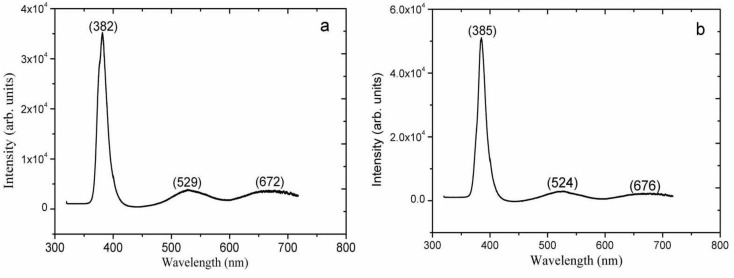
The room temperature PL spectra of ZnO nanorods grown with difference seed layer solution consisting of (**a**) 2.0 mg/mL of ZnO nanoparticles in 3.5 mg/mL of starch concentration; (**b**) 7.5 mg/mL of ZnO nanoparticles in 3.5 mg/mL of cellulose concentration.

A room temperature PL spectroscopy study has been carried out in order to get insight into the optical properties of the ZnO nanorods grown on the biocomposite seed layer containing ZnO nanoparticles. The PL emission of a sample containing ZnO nanorods grown on a seed layer consisting of 2.0 mg/mL of ZnO nanoparticles in 3.5 mg/mL of starch is shown by the spectrum in Figure 9a. Figure 9b shows the PL emission from a sample containing ZnO nanorods grown on a seed layer consisting of 7.5 mg/mL of ZnO nanoparticles in 3.5 mg/mL of cellulose. In both PL spectra, three different emission peaks are observed.

The UV emission is the dominating contribution in both spectra, and has a maximum at 382 nm and 385 nm (3.24 eV and 3.22 eV), for the ZnO sample grown on a starch seed layer and a cellulose seed layer, respectively. These spectral peaks originate from near band edge (NBE) emission, such as recombination of free excitons. In addition to the slight red shift of the NBE emission from the ZnO sample utilizing a cellulose seed layer, the PL intensity is also a bit higher than when starch was used as a seed layer which might be due to possible more defect states in the sample grown with the seed solution of cellulose. The two other peaks found in the PL spectra are very broad and appear at 530 nm and 670 nm (2.3 eV and 1.8 eV). They are attributed to defects in the material.

Green PL emission from ZnO is quite common, and has been observed from ZnO nanorods prepared by low temperature aqueous chemical growth [34,35], vapor-liquid-solid growth [36], CVD [37,38] and the electrochemical growth techniques [39,40]. The green emission is attributed to the recombination of electrons at oxygen vacancies with holes in the valence band [40]. The orange/red emission from ZnO is less well understood. However, oxygen interstitials in the ZnO crystal are possible contributors to this emission [41,42,43,44]. The PL results of the ZnO nanorods sample grown on a starch and cellulose seed layer, respectively, both show defects related emission that is much weaker than the NBE emission, indicating a low concentration of defects, such as oxygen vacancies. Moreover, PL study has described good crystal quality with less defects states in ZnO nanorods using the biocomposite seed layer of ZnO nanoparticles.

## 3. Experimental Section

### 3.1. Preparation of ZnO Nanoparticles Seed Solution in Starch and Cellulose Biopolymers

The seed solution of ZnO nanoparticles without using biopolymers was prepared in 1% acetic acid solution with concentration of 3.5 mg/mL in order to study the role of starch and cellulose on the alignment and orientation of ZnO nanorods. The seed solution of ZnO nanoparticles in starch and cellulose was prepared in a 1% acetic acid solution in an ultrasonic bath for 15 min. The average size of the ZnO particles used as the seed was about 12.2 nm. Different quantities of ZnO nanoparticles, including 0.5, 1.0, 1.5, 2.0, and 2.5 mg/mL were dissolved in the mixture of 10 mL of 1% acetic acid and 3.5 mg/mL of starch. Likewise, seed solution containing 3.5 mg/mL of cellulose and 0, 1.0, 3.5 and 7.5 mg/mL of ZnO nanoparticles was prepared in 10 mL of 1% acetic acid solution. Different concentrations of ZnO nanoparticles were used for monitoring of the effect of nanoparticles on the alignment of nanorods. The seed solutions of starch and cellulose were individually prepared and separately used as seed layer for the fabrication of well aligned ZnO nanorods on the gold-coated glass substrate.

### 3.2. Synthesis of ZnO Nanorods on Gold-Coated Glass Substrates

Prior to growth of ZnO nanorods, glass substrates were coated with 100 nm thickness of gold. The gold coating process began by cleaning the glass substrates with isopropanol in an ultrasonic bath for 20 min. These were then washed with the deionized water and dried in air. Then, the cleaned glass substrates were affixed in the deposition chamber of the evaporator, Satis (725). After achieving vacuum inside the chamber, an adhesive layer of titanium, with a thickness of 20 nm, was deposited on the glass substrate, followed by the deposition of 100 nm thickness of gold. After gold layer deposition, the substrates were cleaned with the deionized water and dried in a nitrogen gas flow. The substrates were then spin coated with the composite seed solution of ZnO nanoparticles and starch two to three times at 3000 r.p.m. for 30 s. Similarly, the gold layer coated substrates were also spin coated with the composite seed solution of ZnO nanoparticles and cellulose. After the seed layer deposition, the substrates were annealed at 120 °C for 20 min. An equimolar solution of 75 mM of zinc nitrate hexahydrate and hexamethylenetetramine (HMT) was prepared in the deionized water and the annealed substrates were fixed in a Teflon sample holder. These were then vertically dipped in the precursor solution and covered with aluminum foil. The growth solution was kept in a preheated oven for four to seven hours at 96 °C. After the completion of growth period, the ZnO nanostructures on gold-coated substrates were washed with the deionized water in order to remove the solid ZnO powder from the surface of the nanostructures, and then dried in air at room temperature.

The crystal structure of ZnO nanoparticles and nanorods was studied by scans (0.1/s) Phillips PW 1729 powder diffractometer using the CuKα radiation (λ = 1.5418 Å). An atomic force microscope (AFM, Dimension 3100) was used for the morphological study of biocomposite seed layer of ZnO nanoparticles. The field emission scanning electron microscopy (FESEM) that was performed using LEO 1550 Gemini, field emission gun was operated at 20 kV for the morphological study of ZnO nanorods and energy-dispersive X-ray (EDX). In the photoluminescence technique a third harmonics (λe = 266 nm) from a Coherent Ti: sapphire laser was used and the detection was performed with Hamamatsu CCD camera. For the dispersion of PL signal a single monochromator of 1 m focal length (model Brucker Optics Chromex 25) is combined with a diffraction grating of 150 lines/mm.

## 4. Conclusions

In this work, well aligned ZnO nanorods were synthesized by hydrothermal growth technique on the gold-coated glass substrates using the biocomposite seed layer of ZnO nanoparticles in starch and cellulose, respectively. The biocomposite seed layer of ZnO nanoparticles has influence on the alignment and diameter of ZnO nanorods. The morphological study of the biocomposite seed layer of ZnO nanoparticles was studied by AFM and the nanoparticles are distributed uniform and well adhered to the surface of gold-coated glass substrate. The ZnO nanorods were characterized by XRD technique, FESEM imaging, and PL spectroscopy and the obtained results show that the nanorods are uniformly distributed with a high density, having well-ordered alignment along the *c*-axis of the substrate, and exhibited good crystal quality. The present approach for the fabrication of ZnO nanorods can be used for the development of improved performance optoelectronic devices such as white LEDs where the rod alignment has a significant effect on the performance of the device.
